# Development and evaluation of a rapid CRISPR-based diagnostic for COVID-19

**DOI:** 10.1371/journal.ppat.1008705

**Published:** 2020-08-27

**Authors:** Tieying Hou, Weiqi Zeng, Minling Yang, Wenjing Chen, Lili Ren, Jingwen Ai, Ji Wu, Yalong Liao, Xuejing Gou, Yongjun Li, Xiaorui Wang, Hang Su, Bing Gu, Jianwei Wang, Teng Xu

**Affiliations:** 1 Laboratory Medicine, Provincial People's Hospital, Guangdong Academy of Medical Sciences Guangzhou, Guangdong, China; 2 Vision Medicals Center for Infectious Diseases, Guangdong, China; 3 Key Laboratory of Animal Gene Editing and Animal Cloning in Yunnan Province and College of Veterinary Medicine, Yunnan Agricultural University, Kunming, China; 4 NHC Key Laboratory of Systems Biology of Pathogens and Christophe Mérieux Laboratory, IPB, CAMS-Fondation Mérieux, Institute of Pathogen Biology (IPB), Chinese Academy of Medical Sciences (CAMS) & Peking Union Medical College, Beijing, China; 5 Department of Infectious Diseases, Huashan Hospital affiliated to Fudan University, Shanghai, China; 6 Medical Technology School of Xuzhou Medical University, Xuzhou, China; 7 Department of Laboratory Medicine, Affiliated Hospital of Xuzhou Medical University, Xuzhou, China; Icahn School of Medicine at Mount Sinai, UNITED STATES

## Abstract

The recent outbreak of human infections caused by SARS-CoV-2, the third zoonotic coronavirus has raised great public health concern globally. Rapid and accurate diagnosis of this novel pathogen posts great challenges not only clinically but also technologically. Metagenomic next-generation sequencing (mNGS) and reverse-transcription PCR (RT-PCR) have been the most commonly used molecular methodologies. However, each has their own limitations. In this study, we developed an isothermal, CRISPR-based diagnostic for COVID-19 with near single-copy sensitivity. The diagnostic performances of all three technology platforms were also compared. Our study aimed to provide more insights into the molecular detection of SARS-CoV-2, and also to present a novel diagnostic option for this new emerging virus.

## Author summary

The recently discovered betacoronavirus severe acute respiratory syndrome (SARS)-CoV-2 has caused an outbreak of Coronavirus Disease 2019 (COVID-19) that rapidly developed into a global pandemic. The surging demand for rapid screening and identification of COVID-19 posts great diagnostic challenges. A lack of rapid and accurate molecular tools has hampered efficient public health responses to the viral threat. Here, we harnessed the unique collateral activity of programmable CRISPR/Cas13a to develop CRISPR-COVID, a rapid and sensitive diagnostic for SARS-CoV-2 infection, and compared it to sequencing-based metagenomic and RT-PCR-based assays in a clinical cohort. CRISPR-COVID demonstrated a sensitivity level of near single copy and was highly specific without cross reacting to related pathogens. CRISPR-COVID takes only 40 mins and requires no sophiscated thermo-cyclers, providing a valuable alternative to the conventional RT–PCR assay to circumvent the bottlenecks in assay turnaround time, equipment and reagent supplies for COVID-19 testing.

## Introduction

Since the beginning of 2020, a surging number of pneumonia caused by infections of a novel coronavirus (SARS-CoV-2) have been identified in China, especially Wuhan, a metropolitan city with a population of over 10 million. [1, 2] In recent days, the outbreak has affected multiple countries and caused worldwide impact.[3–6] Currently, nucleic-acid based tests have been widely used as the reference method for the diagnosis of COVID-19.[7] As of now, over five million individuals have been identified with SARS-CoV-2 infection. As the pandemic develops, there are increasing demands for rapid and sensitive diagnostics for the novel pathogen.

Coronaviruses (CoVs) are positive-sense, single-strand RNA viruses, with four major structural proteins including spike (S), membrane (M), envelop (E) and nucleoprotein (N).[8, 9] Prior to SARS-CoV-2, there were six CoVs that were known to be pathogenic to humans: HCoV-OC43, HCoV-NL63, HCoV-HKU1, HCoV-229E and highly transmissible and pathogenic SARS-CoV and MERS-CoV. [10–12]

The pandemic of COVID-19 was first discovered by metagenomic next-generation sequencing (mNGS) in which the novel virus was found to be a new pathogenic member of the betacoronavirus genus but shared only about 79% in genetic similarity with SARS-CoV.[8, 9] Currently, metagenomics and RT-PCR are two molecular approaches most commonly used diagnostics for this novel virus. [13,14] However, the diagnostic performance of different molecular methods has not been investigated.

Although CRISPR/Cas has been widely used as a programmable tool for gene editing since 2013, the collateral, promiscuous cleavage activities of a unique group of Cas nucleases only discovered recently and harnessed for in vitro nucleic acid detection. [15–19] A CRISPR-based diagnostic was developed for detection of Mycobacterium Tuberculosis and demonstrated comparable sensitivity with GeneXpert assay. [17] Very recently, Broughton *et* Cas12, showing the promising potential of using the CRISPR tools for in vitro diagnostics [20] A tool kit of rapid diagnostics faster than typical RT-PCR is in great demand to circumvent the bottlenecks in assay turnaround time and reagent supply for COVID-19 testing.

Here, to address the expanding clinical needs, we developed CRISPR-COVID, a rapid assay for SARS-CoV-2 detection based on Cas13a, and compared the diagnostic performance among three different technological platforms: metagenomic sequencing, RT-PCR and CRISPR. To our knowledge, this is the first report on cross-platform comparison and the evaluation of an isothermal, CRISPR-based assay for COVID-19 that's rapid, sensitive and with low instrument requirement.

## Results

### Identification of COVID-19 by mNGS

In order to develop a targeted assay for the novel virus, we obtained total RNA samples from 61 cases suspected for COVID-19 and subjected them to metagenomic next-generation sequencing (mNGS), the method by which this novel virus was initially identified.

**Fig 1:**
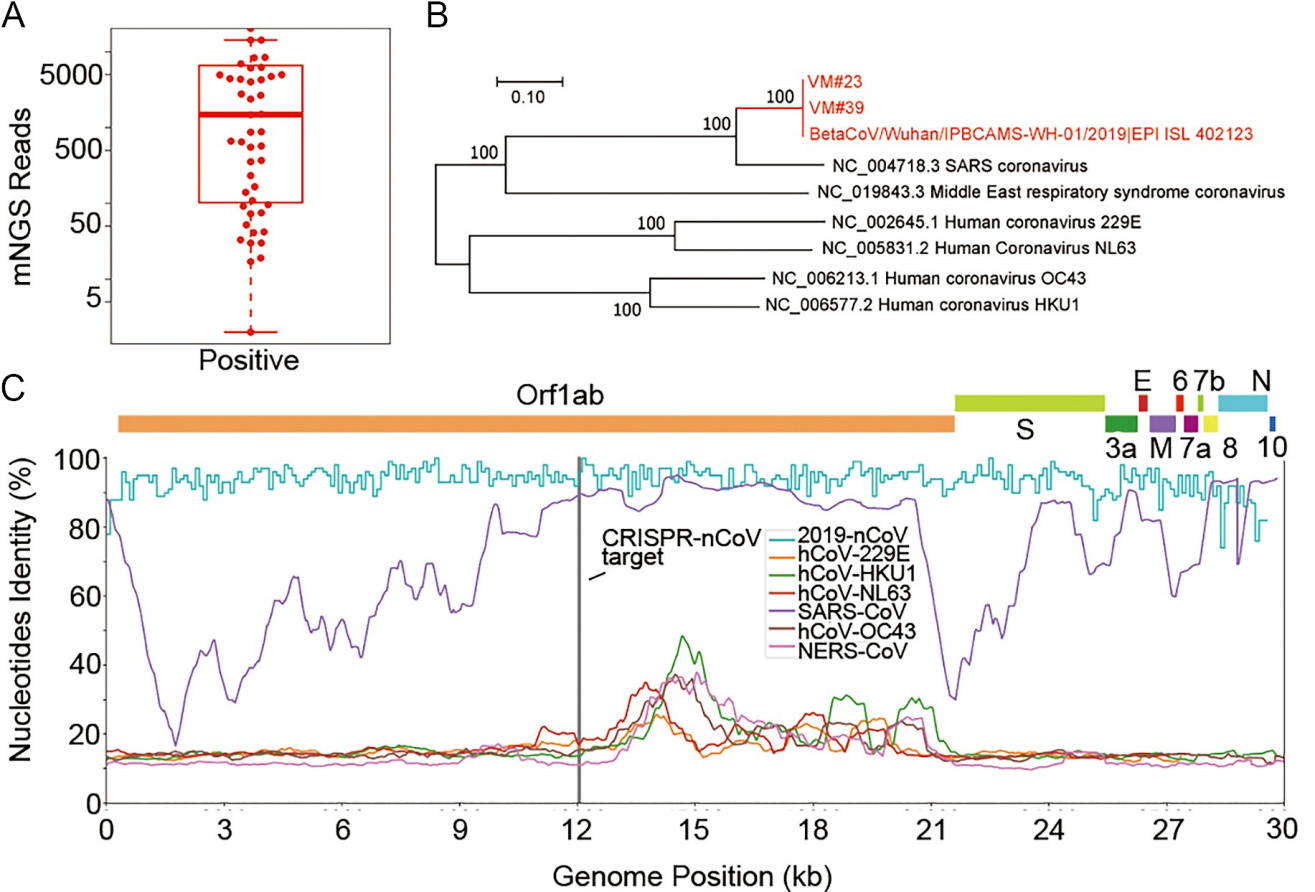
**(A)** Number of mNGS reads mapped to the genome of SARS-CoV-2 in 52 positive cases; **(B)** Phylogenetic tree of SARS-CoV-2s and other pathogenic CoVs; **(C)** SARS-CoV-2 genome and CRISPR target region. Nucleotide identity among the SARS-CoV-2 genomes, including 52 in our cohort and 2,439 from public databases (Blue), between SARS-CoV-2 (NC_045512.2) and other pathogenic CoVs (Red, SARS-CoV; others as indicated). Brief gene locations are presented above and the target region for CRISPR-COVID is indicated in Grey.

Among the 61 suspected COVID-19 specimens, we were able to confirm 52 cases with read numbers mapping to the novel virus across 6 orders of magnitudes (median read of 1,484, from 2 to 19,016,501). The median genome coverage and sequencing depth was 46.8% (2.8%-100%) and 12.0× (1.0×-7870.1×), respectively (Fig 1A and S1 Fig). These findings indicate a high degree of variation in viral loads of SARS-CoV-2. As shown in the phylogenetic trees, SARS-CoV-2 genome identified in our specimens were highly conserved with the Wuhan strain and closest to SARS-CoV (Fig 1B).

With this genomic information, we aimed to identify target regions of SARS-CoV-2 by searching for sequences that were i) within the *Orf1ab*, *N* or *E* genes of the viral genome; ii) conserved among strains of the novel virus; iii) differentiable from other pathogenic coronaviruses. By analyzing the genetic similarity among the SARS-CoV-2 genomes and other pathogenic CoVs (Fig 1C), we identified two potential target sequences in *Orf1ab* and one in the *N* gene.

### Development and evaluation of CRISPR-COVID

**Fig 2:**
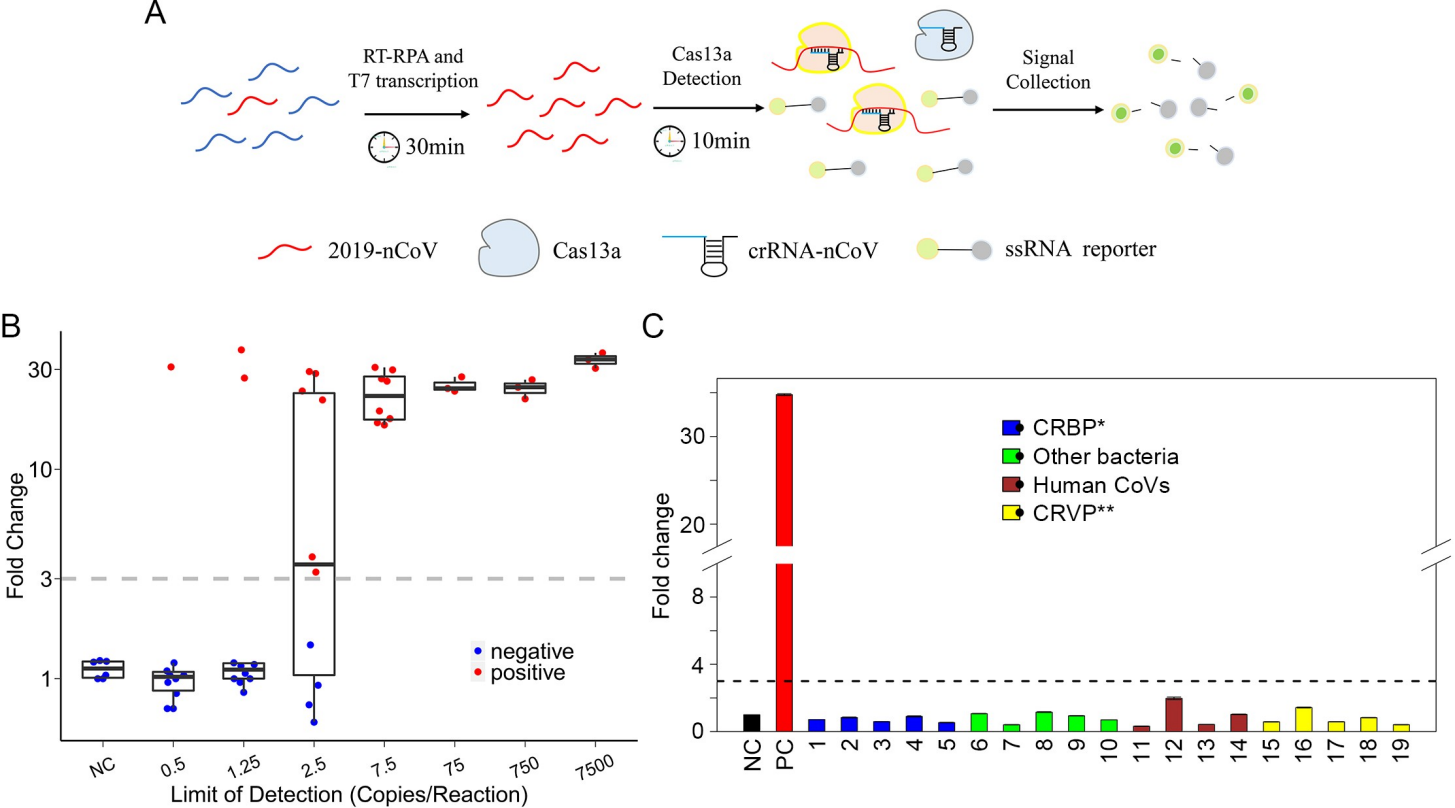
Analytical assessment of the sensitivity and specificity of CRISPR-COVID. **(A)** Schematic diagram of CRISPR-COVID. The collateral nuclease activity of Cas proteins is activated upon specific binding of gRNA to the Orf1ab gene. Fluorescent signal produced from cleaved probes is captured and indicates the presence of SARS-CoV-2; Analytic evaluation of assay performance by testing contrived negative swab samples with indicated titers of SARS-CoV-2 **(B)**, and various microbes as interfering materials. (CRBP, Common respiratory bacterial pathogens; CRVP, Common respiratory viral pathogens; 1, *S*. *pneumoniae*; 2, *H*. *influenzae*; 3, *M*. *pneumoniae*; 4, *C*. *pneumoniae*; 5, *B*. *pertusiss*, 6, *S*. *mitis*; 7, *S*. *pyogenes*; 8, *S*. *aureus*; 9, *E*. *coli*; 10, *E*. *faecalis*; 11, *hCoV-OC43*; 12, *hCoV-NL63*; 13, *hCoV-HKU-1*; 14, *hCoV-229E*; 15, *Adenovirus Type 3*; 16, *H*. *influenzae B (Victoria)*; 17, *H*. *influenzae A (H3N2)*; 18, *HPIV-1*; 19, *RSV-A*) **(C)**. Group Blue, common respiratory bacterial pathogens; Green, Other bacteria; Brown, other CoVs; Yellow, common respiratory viral pathogens. P < 0.001 by Student's t-test between PC and all other samples.

We seek to develop a rapid, highly sensitive and simple-to-use assay by taking advantage of both the polymerase-mediated DNA amplification by RPA and the CRISPR/Cas-mediated enzymatic signal amplification for improved sensitivity (Fig 2A). Moreover, the isothermal nature of such an assay abolished the demand for sophiscated instruments such as thermal cyclers as for PCR-based assays.

Based on the three potential target sequences we identified, multiple sets of RPA primers and CRISPR gRNAs were designed and screened. Among these, the set that targeted *Orf1ab* showed the best overall performance of sensitivity and specificity, and therefore, was used to develop CRISPR-COVID in this study for further evaluation (S2A, S2B and S2C Fig).

We then sought to determine its analytic sensitivity by serial dilution at various concentrations. As shown in Fig 2B, CRISPR-COVID consistently detected 7.5 copies/reaction of SARS-CoV-2 in all 10 replicates, 2.5 copies/reaction in 6 out of 10, and 1.25 copies/reaction in 2 out of 10 runs. These data indicate that CRISPR-COVID had a near single-copy sensitivity. To confirm its specificity, we tested CRISPR-COVID with DNA from human cells as well as a panel of microbes including i) bacteria commonly found in respiratory infections: *S*. *pneumonia*, *H*. *influenza*, *M*. *pneumonia*, *C*. *pneumonia*, *B*. *pertusiss;* ii) human Coronaviruses: *HCoV-OC43*, *HCoV-NL63*, *HCoV-HKU1*, *HCoV-229E;* iii) other viruses commonly found in respiratory infections: *Adenovirus Type-3*, *H*. *Influenza B (Victoria)*, *H influenza A (H3N2)*, *HPIV-1*, *RSV-A*; and iii) other bacteria: *S*. *mitis*, *S*. *pyogenes*, *S*. *aureus*, *E*. *coli*, *E*. *facecaiis*. None of the above interference samples triggered a false positive reaction (Fig 2C). Altogether, these analytical assessments suggest CRISPR-COVID as a promising molecular assay for SARS-CoV-2 detection with great sensitivity and specificity.

**Fig 3:**
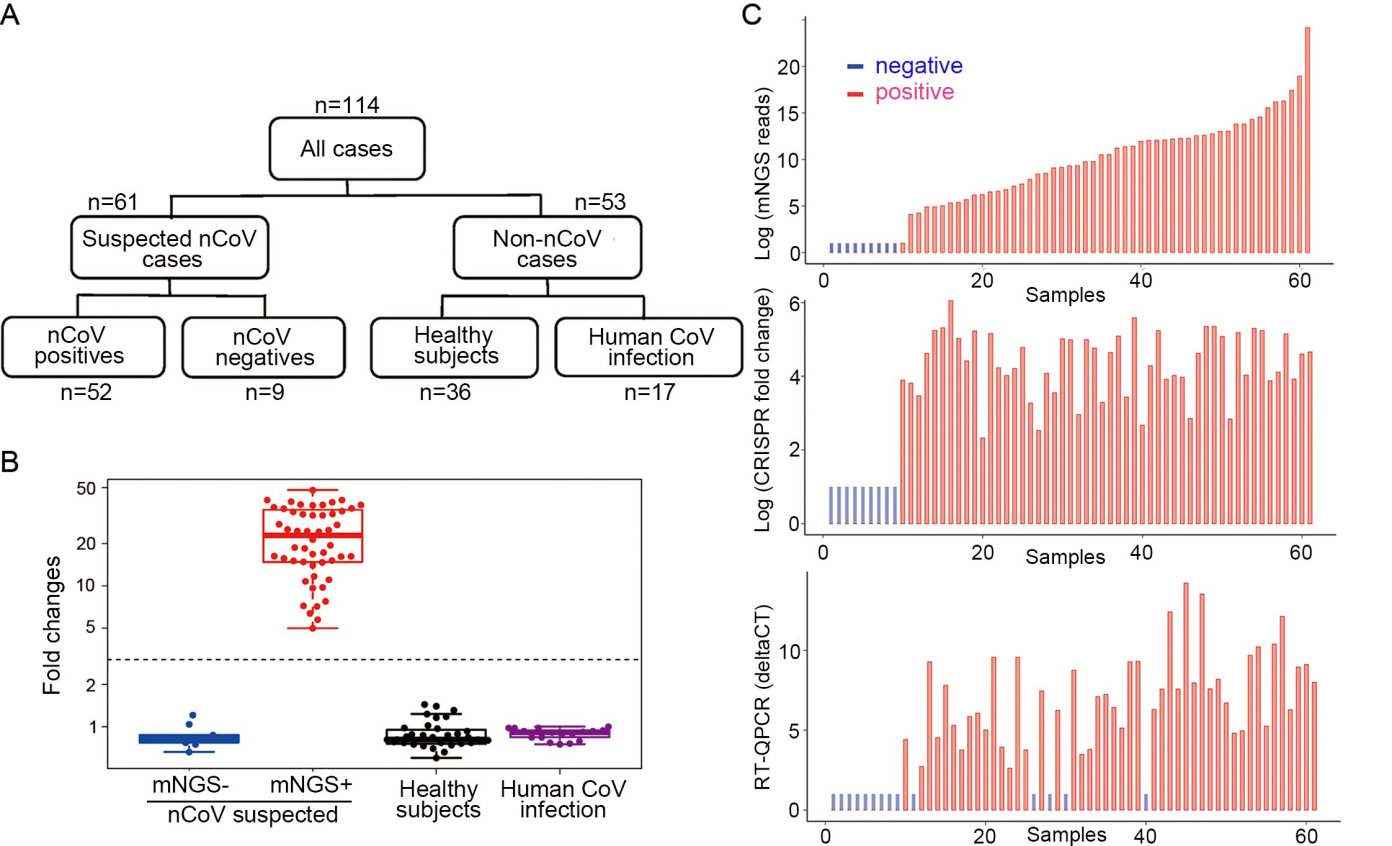
Summary of the cohort and CRISPR-COVID results. **(A**) A total of 112 specimens included in this study, including 52 cases positive for SARS-CoV-2 and 62 negative cases. **(B)** Results of CRISPR-COVID in different sample groups. Positives and negatives were called based on the fold change and cutoff values; **(C)** Result summary on the suspected SARS-CoV-2 samples by mNGS, CRISPR-COVID and PCR-COVID. P = 0.056 between mNGS and RT-PCR, and between CRISPR and RT-PCR by Fisher's exact test.

Upon completion of the analytical assessment, we further evaluated the diagnostic potential of CRIPSR-COVID in clinical specimens. A total of 114 RNA samples from clinical respiratory samples were included in the evaluation, which consisted of 61 suspected COVID-19 cases (among which 52 confirmed and 9 ruled-out by mNGS), 17 SARS-CoV-2-/hCoV+ cases and 36 samples from healthy subjects (Fig 3A).

When conducting the CRISPR-COVID assay, a positive control (PC) DNA and a no-template control (NC) were included in parallel for each run. Florescent signal from NC was used to normalize the signal and generate corresponding fold-change values (FC). We noticed that there was a clear distinction in signal patterns of the reactions (S3A Fig). Specifically, the fluorescent signal curve either remained flat as a negative curve (e.g. the NC runs) or had a distinguishable positive signal curve (e.g. the PC runs). The negative curves yielded a maximal FC value of 1.4, whereas the positive ones had a minimal FC value of 5.0. A cut-off of 3.0 was set for optimal readout separation and assay performance as confirmed by an ROC analysis (S3B and S3C Fig).

CRISPR-COVID demonstrated a sensitivity of 100% by detecting all 52 COVID-19 cases. No false positives were found in all 62 negative cases, including all the hCoV-infected ones (Fig 3B), suggesting promising clinical sensitivity and specificity of CRISPR-COVID. To further evaluate assay compatibility, the primer and gRNA sequences used in CRISPR-COVID were aligned to 2,439 SARS-CoV-2 genomes obtained from public databases. We found vast majority of these genomes (99.63%, 2,430/2,439) to be free of mutations in the binding regions. Only 0.16% (4/2,439) harbored mutations in the 3’ end of the primer binding sites that would potentially affect assay performance (S4 Fig). As these genomes covered wide ranges of collection times (from 2019/12/24 to 2020/05/06) and geographic locations (over 20 countries including China, United States and France etc.), our results suggest the general applicability of our assay for COVID-19 diagnosis.

### Diagnostic Performances Across Technology Platforms

We further set out to compare the diagnostic performances among mNGS, RT-PCR and CRISPR. Using mNGS and the PCR as the reference, CRISPR-COVID had a specificity of 100% in our study. PCR-COVID was able to detect the virus in 90.4% (47/52) of the positive cases, with Ct’s ranging from 28.8 to 40.4 and a median Ct of 35.8. It’s worth noting that the 5 false negative samples by PCR-COVID had a median mNGS read number of 550, which was much lower than that of the other positive samples at 2,381 reads, suggesting a lower titer of the virus in these samples. CRISPR showed a greater sensitivity by detecting all 52 COVID-19 cases (100%), with FC values ranging from 5.0 to 66.3 and a median FC of 22.8 (Fig 3C).

**Fig 4:**
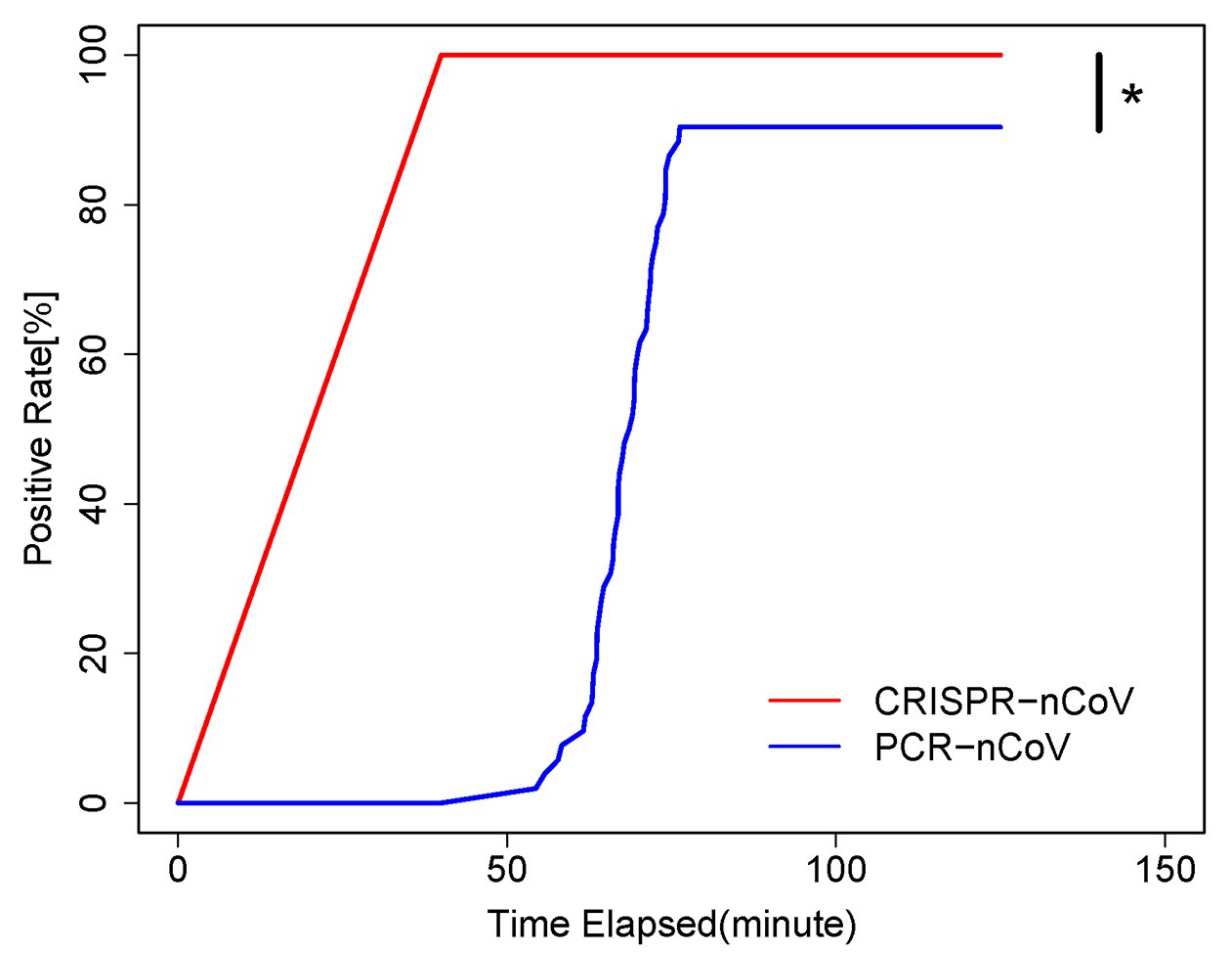
Kaplan–Meier curve of CRISPR-COVID positive rate by CRISPR and PCR. * Log-rank test, P < 0.05.

When the reaction turn-around time (TAT) is compared, the CRISPR-COVID reaction requires only 40 minutes, which is the least among the three and includes 30 minutes of DNA amplification and 10 minutes of Cas reaction. PCR-COVID requires about 1.5 hours for a completion run of the PCR program. mNGS takes approximately 20 hours, which includes 8 hours of library preparation, 10 hours of sequencing and 2 hours of bioinformatic analysis. Because a positive result may be determined before completing the PCR program, we calculated the effective TAT as the time when the florescent signal reached the threshold. As showed in Fig 4, CRISPR-COVID presented a significant advantage in effective TAT over PCR and mNGS.

Altogether, we demonstrated a CRISPR-based assay for COVID-19 that offered shorter turn-around time and great diagnostic value, even in under-resourced settings without the need of thermal cyclers. Our study also emphasized strength and weakness of different methodologies which should be fully considered when applied in different diagnostic settings.

## Discussion

Recent progresses in molecular diagnostic technologies, especially mNGS, allowed rapid, initial identification of this novel pathogenic agent at the beginning of the current COVID-19 pandemic.[8] Through acquiring the genome sequences of the novel virus, RT-PCR assays were quickly developed for targeted SARS-CoV-2 detection.[14] However, the sudden outbreak of COVID-19 created a dramatic burden not only on the society, but also on public health. The center of this epidemic, Wuhan city alone, hosts a population of over 10 million. The surging demand for rapid screening and identification of COVID-19 posts a great challenge on the diagnostics.[21] Sequencing is the method originally used for the identification of this new viral species and considered as one of the most important references.[8, 22] However, its wider application is limited by its cost and longer TAT of nearly a day. An RT-PCR assay for COVID-19 is faster and more affordable. Nevertheless, the need for a thermo cycler by PCR-based diagnostics hinders its use in low-resource settings and curbs the assay throughput. Besides their lower demand for sophisticated temperature controlling instruments, isothermal molecular methods are advantageous owing to its faster nucleic acid amplification. However, there have been debates over the specificity of such methods. The current discovery of the collateral activity of certain Cas family members, provides a great opportunity to take advantage of both the sensitivity of an isothermal assay and the specificity of the CRISPR system [19]. As we demonstrated in this study, CRISPR-COVID was able to deliver comparable sensitivity and specificity as mNGS within as short as 40 minutes. At research scale, the material costs of a CRISPR-COVID test runs at less than $3.5, which could be dramatically reduced at production scale to below $0.7[23], suggesting CRISPR-COVID as a competitive alternative not only technologically but also financially.

As a result of the rapid outbreak, the targeted assays (PCR and CRISPR) were designed and developed based on limited genetic information on SARS-CoV-2. Cautions should be taken that certain unknown genomic variations may produce critical impact on the assay efficiency. For example, a mutation or polymorphic site that abrupt the binding of the very 3’ end of a PCR primer may cause a drastic reduction in sensitivity. As the CRISPR-COVID assay uses similar sample collection and extraction methods as other nucleic acid-based assays, similar limitations were shared in the availability of personal protective equipment and extraction reagents. However, the key advantages of isothermal reaction and short turn-around of CRISPR-COVID allows fast and high-throughput testing with lower demands in laboratory requirements. Although not without its own shortcomings on TAT and costs, NGS-based assays demonstrated a great level of sensitivity and should still be used for continuous monitoring of genetic drifts in the viral genome. This information will provide valuable insights not only from a public health standpoint, but also to guide necessary optimization of targeted assay development.

## Materials and methods

### Study participants and sample collection

This study used excess RNA samples from patients with suspected SARS-CoV-2 infection based on clinical, chest imaging and epidemiological evidence. No patient identifiable information was collected. The only data collected from the samples were types of specimens (13 nasopharyngeal swab and 39 bronchoalveolar lavage fluid specimens), the concentrations and volumes of the purified total RNA. A total of sixty-one suspected COVID-19 samples were included in this study. Among which, 52 was confirmed positive by mNGS. Non-COVID-19 cases were samples not suspected for COVID-19 based on the clinical and epidemiologic criteria, and ruled out for SARS-CoV-2 infection by mNGS or RT-PCR.

### Ethics statement

This study was approved by the ethical review committee of Institute of Pathogen Biology, Chinese Academy of Medical Sciences & Peking Union Medical College. Written informed consent was waived given the context of emerging infectious diseases.

### RT-PCR and mNGS Assay for COVID-19

RNA was isolated with the QIAamp ViralRNA Mini kit (Qiagen, Valencia, CA). RT-PCR assays for COVID-19 were performed using a clinically validated kit approved by the Chinese National Medical Products Administration (Liferiver, Shanghai, China) on a ABI-7500 Real-Time PCR System (Thermo Fisher Scientific, Carlsbad, CA) according to the instructions, with Ct values below 40 considered positive. For mNGS sequencing, and measured by a Qubit Fluorometer (Thermo Fisher Scientific, Carlsbad, CA). Reverse transcription was performed with N_6_ random primers prior to adaptor ligation with T4 ligase and library amplification, which produced library with fragment sizes ranging from 300-500bp. Sequencing was performed on a NextSeq sequencer (Illumina, San Diego, CA). At least 10 million single-end 75bp reads were generated for each sample. Quality control processes included removal of low-complexity, low-quality, and short reads, as well as adapter trimming. Reads derived from the host genome were then removed. Clean reads were aligned against the reference microbial databases including archaea, bacteria, fungi, protozoa, and viruses. Taxonomic classification of SARS-CoV-2 was determined by reads specifically mapped to the reference genome of SARS-CoV-2 with over 90% in nucleotide identity. Further sequence analyses in mNGS+/PCR- samples were performed to rule out false positive identification caused by cross mapping to other coronaviruses (S5 Fig). A negative control sample was processed and sequenced in parallel for each sequencing run for contamination and background control.

### Phylogenetic analysis

Phylogenetic trees were constructed based on the genome sequences by means of the maximum-likelihood method with 1,000 bootstrap replicates. Alignment of multiple sequences was performed with the ClustalW program (MEGA software, version 7.0.14).

### Cas13a protein and other reagents

The open reading frame (ORF) of Cas13a was synthesized after codon optimization. The Cas13a ORF was then cloned into expression vector Pc013 and transfected into E. coli BL21, which were first grown at 37°C and incubated with IPTG at 16°C. Proteins were purified from lysed bacteria using the Ni-NTA protocol. Aliquots of purified protein were stored at −80°C. Other reagents were purchased from Sangon Co., Ltd. (Shanghai, China), including DTT (A100281), EDTA (A100105), TritonX-100 (A110694), NP-40 (A100109), Chelex-100 (C7901) etc.

### Strains and human DNA

Pure human DNA were purchased from Solarbio Co.,Ltd. (Beijing, China), and eluted in nuclease-free water. *Bacterial and viral* strains were purchased from the American Type Culture Collection (ATCC), China General Microbiological Culture Collection Center (CGMCC) or BDS (Guangzhou, China).

### Oligos and gRNA

Primer with an appended T7 promoter used in the RPA amplification for *Orf1ab* amplification were forward primer 5’-TAAT ACGA CTCA CTAT AGGG ACAT AAAC AAGC TTTG TGAA GAAA TGCT GGAC-3’ and reverse primer 5’-TTGA GCAG TAGC AAAA GCTG CATA TGAT GGAA GG-3’. gRNA for *Orf1ab* (5’-GGGG AUUU AGAC UACC CCAA AAAC GAAG GGGA CUAA AACA AACU CUGA GGCU AUAG CUUG UAAG GUU-3’) and ssRNA probe (5´-6-FAM-UUUU UC-BHQ1) were used for the CRISPR detection following RPA amplification.

### CRISPR-COVID

The CRISPR-COVID test combines an Reverse-transcription Recombinase Polymerase Amplification (RT-RPA) step and a following T7 transcription and Cas13 detection step as described previously. [17] Briefly, reactions containing 2.5 μl of sample, 0.4 μM of each primer and reaction buffer including 50 mM of Tris (pH 7.9), 100 mM of potassium acetate, 14 mM of magnesium acetate, 2 mM of DTT, 200 μM of dNTPs, 3 mM of ATP, 50 mM of phosphocreatine and 14 mM of magnesium acetate, and the RT-RPA enzyme mix were incubated at 42°C for 30 min. After that, the CRISPR reaction mix consisting of the amplification product, 33.3 nM of gRNA, 66.7 nM of Cas13, 5 mM of each NTP, 1μl T7 RNA polymerase (New England Biolabs) and 166 nM of ssRNA reporter was incubated at 42°C and monitored for fluorescence signal. Fluorescent signals were collected for the duration of 10 min.

### Plasmid construction

A 420 bp genomic fragment of *Orf1ab* encompassing 310 bp upstream and 82bp downstream of the gRNA target site was synthesized and inserted into pUC57. These sequences represented 11788–12207 bp in the SARS-CoV-2 genome. This *Orf1ab* plasmid was purified and used as positive control.

### Evaluation of limit of detection (LoD)

For the evaluation of LoD by the number of DNA copies, DNA of the SARS-CoV-2 plasmid was purified and the concentration was determined by a Qubit (Thermo Fisher, Massachusetts). The copy number concentration was then calculated based the weight and the length of fragment. Serial dilution with nuclease-free water was done to achieve desired concentrations. 2.5 μl of extracted DNA at each titer was used as templates. Ten replicates were performed at each data point near detection limit.

### Statistical analysis

Comparative analysis was conducted by Fisher’s exact test, the Student’s t-test or log-rank test where appropriate. Data analyses were performed using SPSS 22.0 software. P values <0.05 were considered significant, and all tests were 2-tailed unless indicated otherwise.

## Supporting information

S1 Table Excel spreadsheet containing, in separate sheets, the underlying numerical data for Figure panels 2b, 2c, 2e, 3d, 3e, 4b, 4c, 4e, 4h, 4h′, 5b, 5d, 5e, 5f, 5h, 5k, 6c, 6d, 6e, 6f, 8c, and 8f. (XLSX)

S1 Fig Genome coverage (left panel) and sequencing depth (right panel) of SARS-CoV-2 in the 52 mNGS+ cases.
(TIF)

S2 Fig Pilot study screening for CRISPR gRNA and primer sets. **(A)** Three different gRNAs targeting N and Orf1ab genes were screened with a positive control to compare for signal production indicated by fold changes in flourescence. **(B, C)** Analytic LoD assessment in top 3 primer sets selected using contrived negative swab samples with indicated titers of SARS-CoV-2 excluding the one used in the final CRISPR-COVID assay. **, P<0.01, student's t-test.
(TIFF)

S3 Fig Determination of assay cut-off. **(A)** Representative signal curves produced by CRISPR-COVID. A positive control (red), a negative control (black) and clinical samples (blue) were shown with distinct positive or negative curve patterns. **(B)** Fold-change values by CRISPR-COVID obtained from our prospective cohort. Positive, i.e. ones with take-off signal curves were in red; Negative, i.e. ones with flat curves were in blue. A cut-off of 3,0 was set and in indicated in black dashed line. **(C)** ROC analysis for cut-off determination.
(TIFF)

S4 Fig Mutations in SARS-CoV-2 genomes and their potential impact on CRISPR-COVID. (TIFF)

S5 Fig Comparison of sequence identifies of potential SARS-CoV-2 reads between SARS-CoV-2 and other coronaviruses known to infect humans in five mNGS+/PCR- cases. ***, P < 0.001, Student’s t-test.
(TIF)
