# Supplementary figures and images for: Development and evaluation of a rapid CRISPR-based diagnostic for COVID-19
Source: PLoS Pathog. 2020 Aug 27;16(8):e1008705. doi: 10.1371/journal.ppat.1008705 (PMC7451577; doi:10.1371/journal.ppat.1008705)

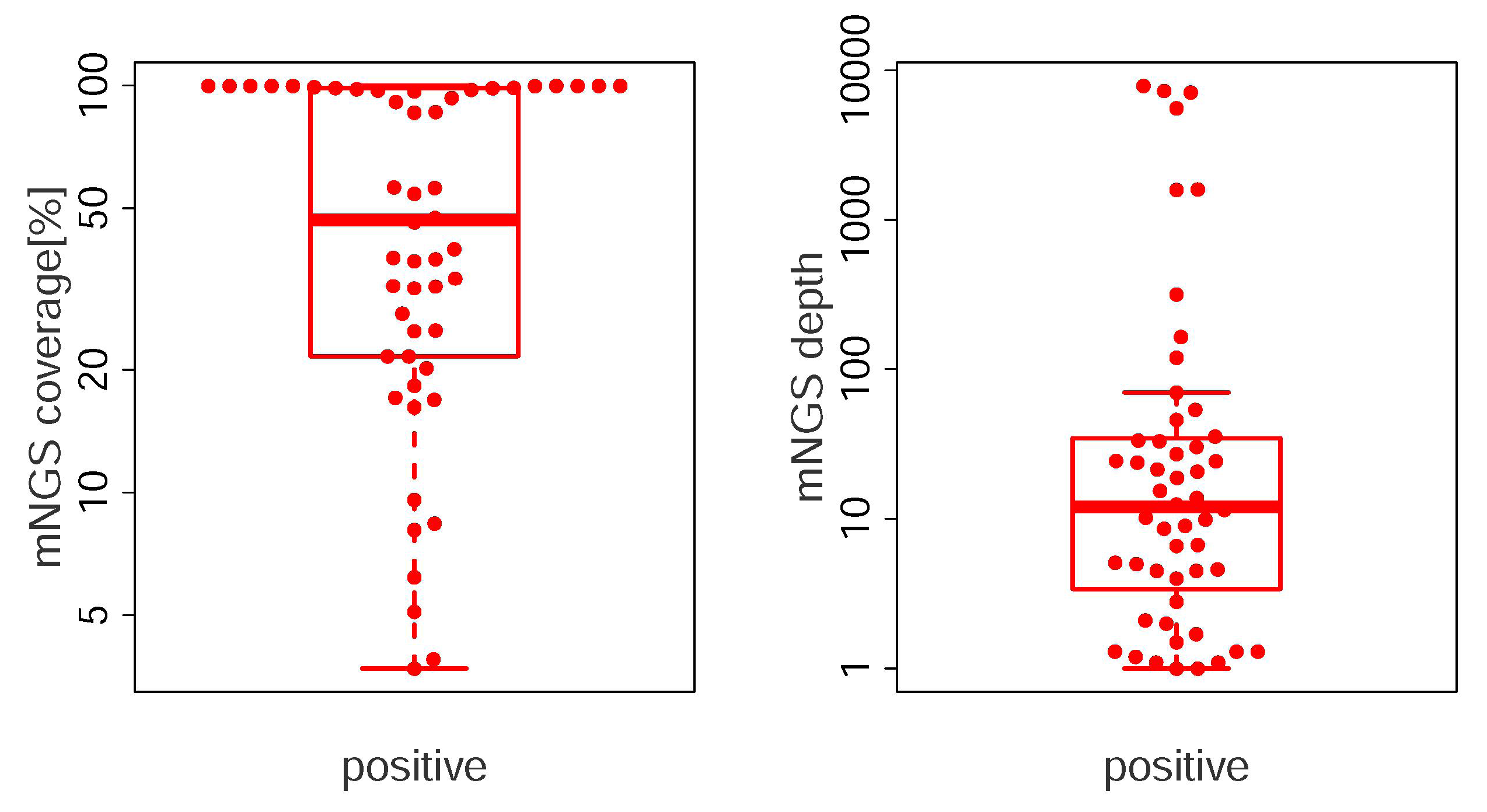

Supplement: S1 Fig — Genome coverage (left panel) and sequencing depth (right panel) of SARS-CoV-2 in the 52 mNGS+ cases. (TIF) [file ppat.1008705.s002.tif]

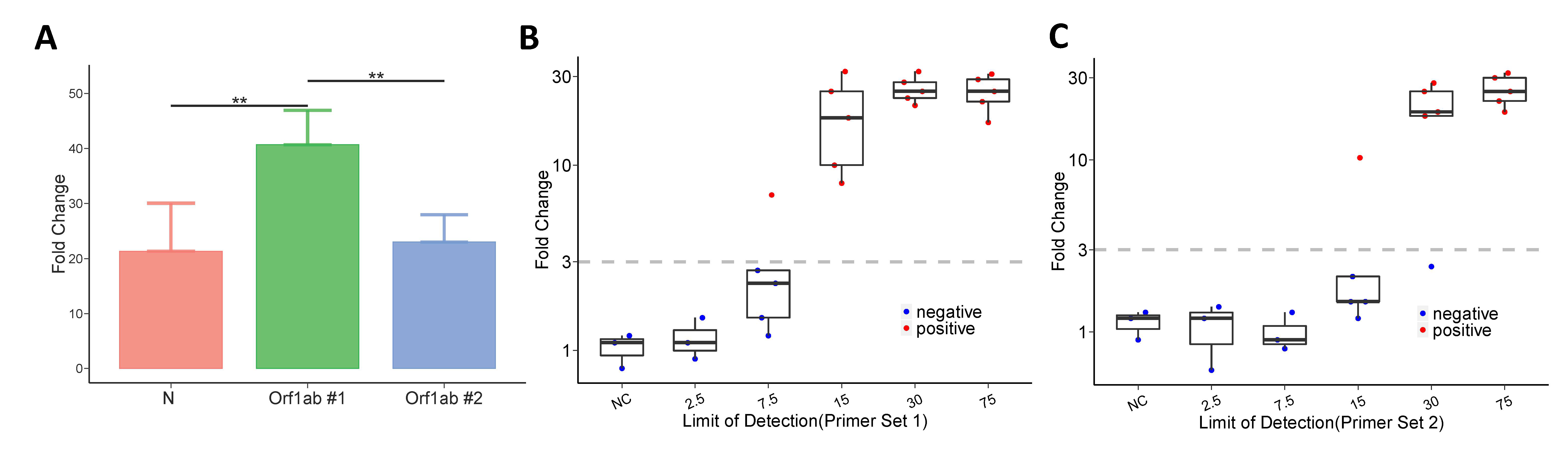

Supplement: S2 Fig — (A) Three different gRNAs targeting N and Orf1ab genes were screened with a positive control to compare for signal production indicated by fold changes in flourescence. (B, C) Analytic LoD assessment in top 3 primer sets selected using contrived negative swab samples with indicated titers of SARS-CoV-2 excluding the one used in the final CRISPR-COVID assay. **, P<0.01, student's t-test. (TIFF) [file ppat.1008705.s003.tiff]

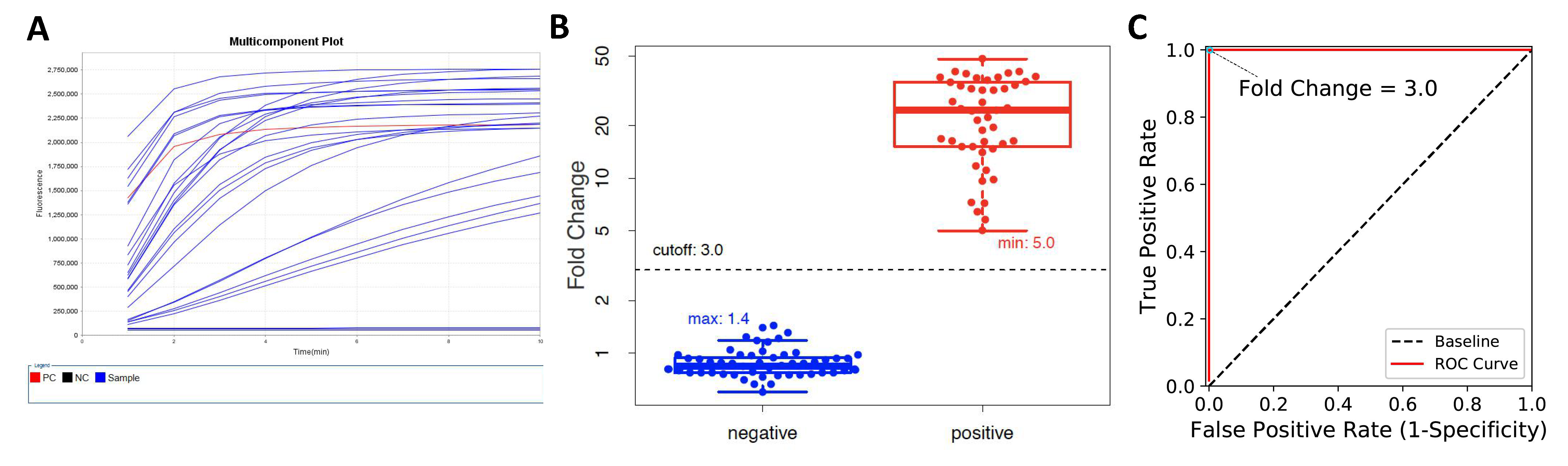

Supplement: S3 Fig — (A) Representative signal curves produced by CRISPR-COVID. A positive control (red), a negative control (black) and clinical samples (blue) were shown with distinct positive or negative curve patterns. (B) Fold-change values by CRISPR-COVID obtained from our prospective cohort. Positive, i.e. ones with take-off signal curves were in red; Negative, i.e. ones with flat curves were in blue. A cut-off of 3,0 was set and in indicated in black dashed line. (C) ROC analysis for cut-off determination. (TIFF) [file ppat.1008705.s004.tiff]

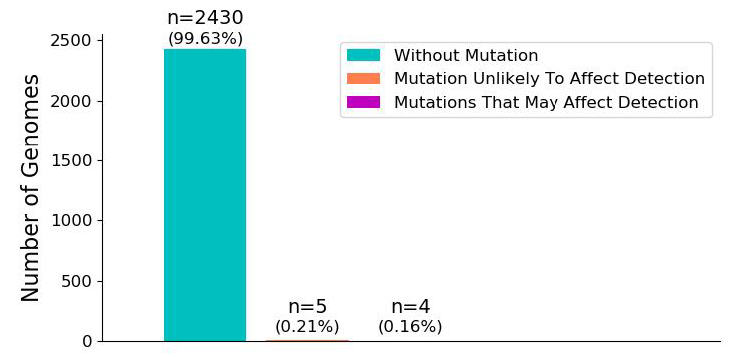

Supplement: S4 Fig — (TIFF) [file ppat.1008705.s005.tiff]

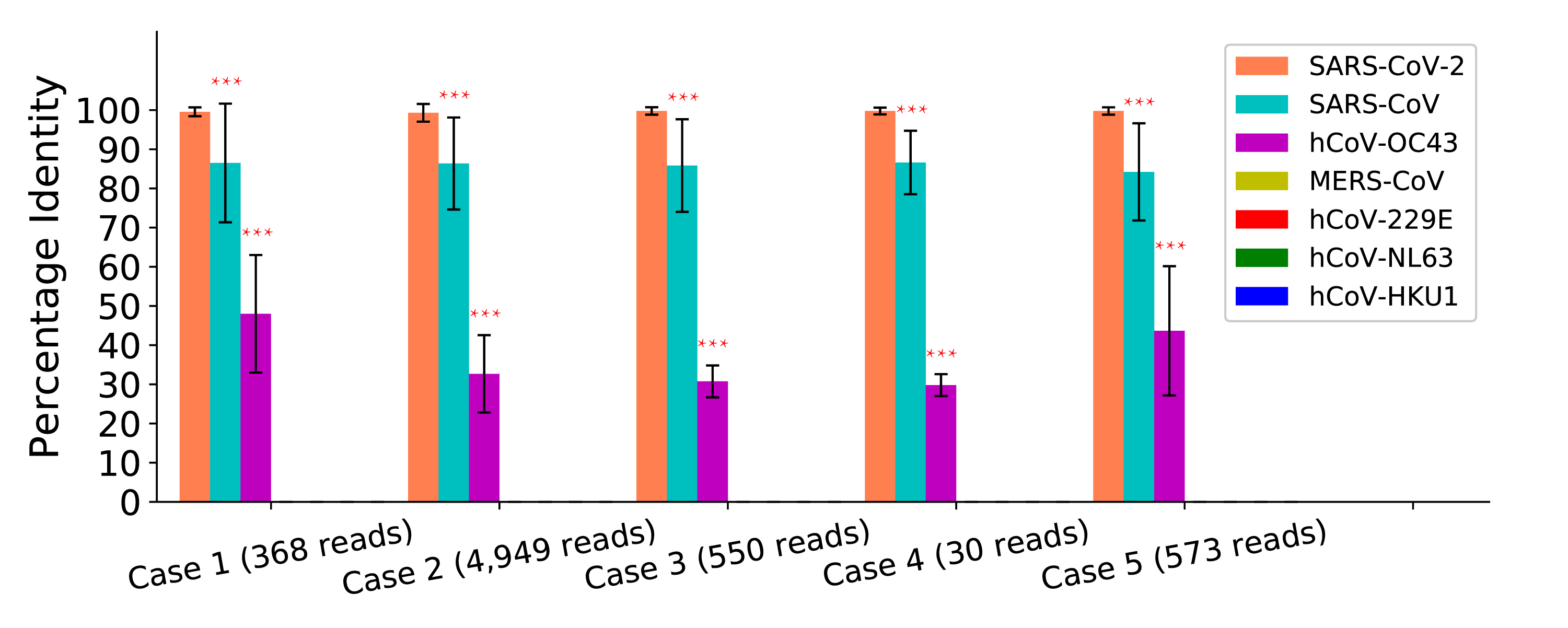

Supplement: S5 Fig — ***, P < 0.001, Student’s t-test. (TIF) [file ppat.1008705.s006.tif]
